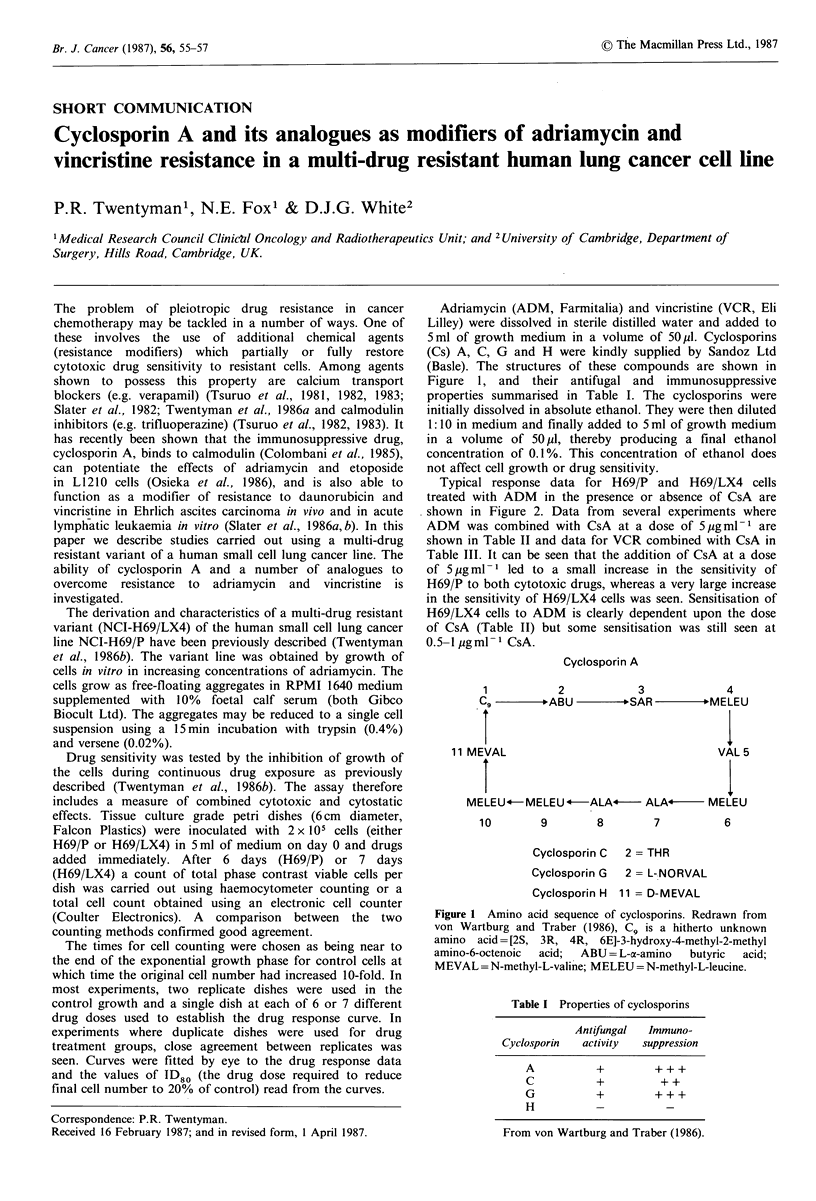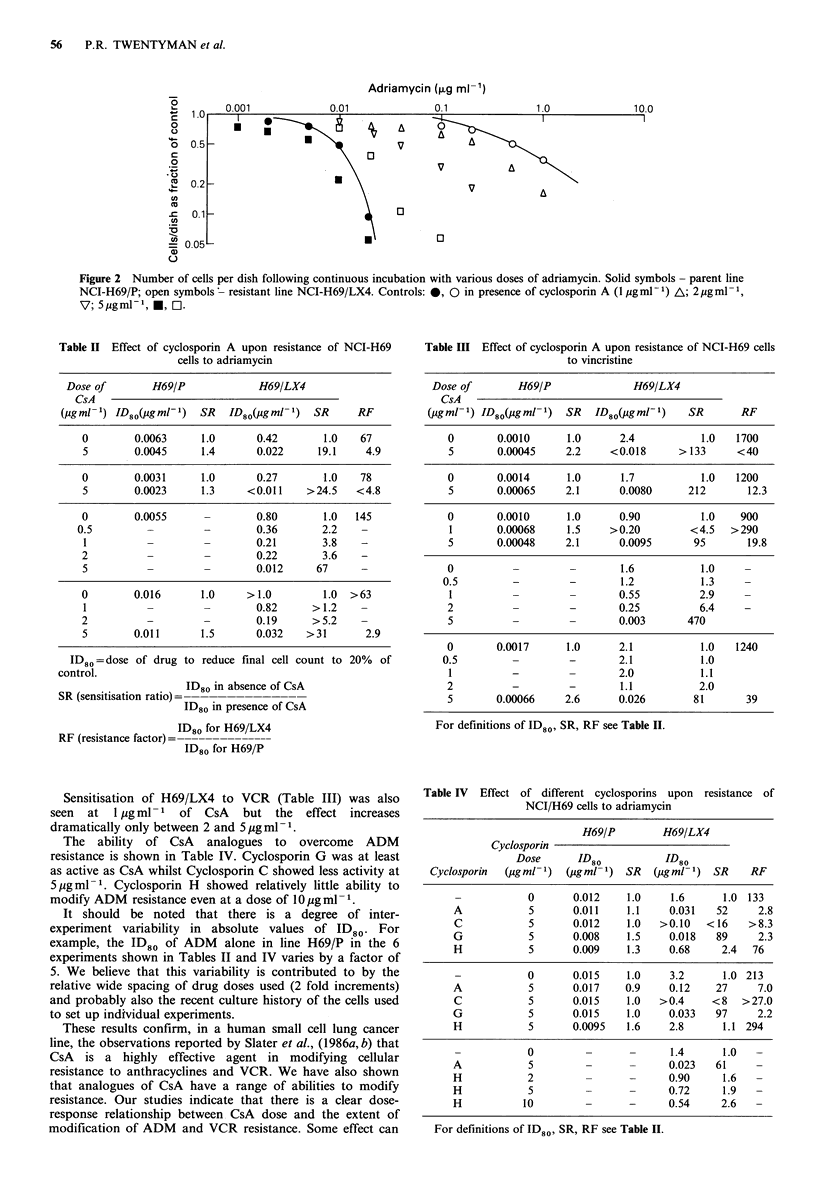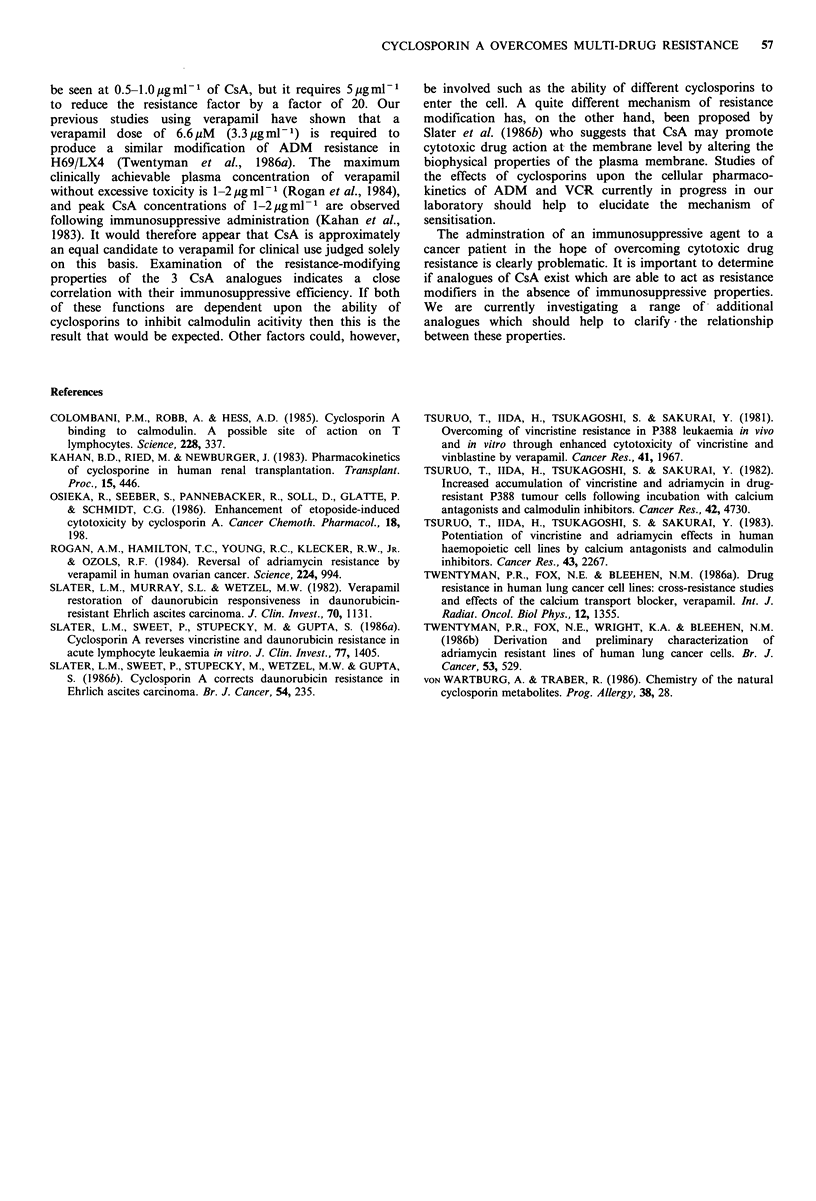# Cyclosporin A and its analogues as modifiers of adriamycin and vincristine resistance in a multi-drug resistant human lung cancer cell line.

**DOI:** 10.1038/bjc.1987.153

**Published:** 1987-07

**Authors:** P. R. Twentyman, N. E. Fox, D. J. White


© The Macmillan Press Ltd., 1987

SHORT COMMUNICATION

### Cyclosporin A and its analogues as modifiers of adriamycin and vincristine resistance in a multi-drug resistant human lung cancer cell line

P.R. Twentyman[1], N.E. Fox[1] & D.J.G. White[2]

[1]*Medical Research Council Clinical Oncology and Radiotherapeutics Unit; and* [2]*University of Cambridge, Department of Surgery, Hills Road, Cambridge, UK.*

The problem of pleiotropic drug resistance in cancer chemotherapy may be tackled in a number of ways. One of these involves the use of additional chemical agents (resistance modifiers) which partially or fully restore cytotoxic drug sensitivity to resistant cells. Among agents shown to possess this property are calcium transport blockers (e.g. verapamil) (Tsuruo *et al.*, 1981, 1982, 1983; Slater *et al.*, 1982; Twentyman *et al.*, 1986*a* and calmodulin inhibitors (e.g. trifluoperazine) (Tsuruo *et al.*, 1982, 1983). It has recently been shown that the immunosuppressive drug, cyclosporin A, binds to calmodulin (Colombani *et al.*, 1985), can potentiate the effects of adriamycin and etoposide in L1210 cells (Osieka *et al.*, 1986), and is also able to function as a modifier of resistance to daunorubicin and vincristine in Ehrlich ascites carcinoma *in vivo* and in acute lymphatic leukaemia *in vitro* (Slater *et al.*, 1986*a*, *b*). In this paper we describe studies carried out using a multi-drug resistant variant of a human small cell lung cancer line. The ability of cyclosporin A and a number of analogues to overcome resistance to adriamycin and vincristine is investigated.

The derivation and characteristics of a multi-drug resistant variant (NCI-H69/LX4) of the human small cell lung cancer line NCI-H69/P have been previously described (Twentyman *et al.*, 1986*b*). The variant line was obtained by growth of cells *in vitro* in increasing concentrations of adriamycin. The cells grow as free-floating aggregates in RPMI 1640 medium supplemented with 10% foetal calf serum (both Gibco Biocult Ltd). The aggregates may be reduced to a single cell suspension using a 15 min incubation with trypsin (0.4%) and versene (0.02%).

Drug sensitivity was tested by the inhibition of growth of the cells during continuous drug exposure as previously described (Twentyman *et al.*, 1986*b*). The assay therefore includes a measure of combined cytotoxic and cytostatic effects. Tissue culture grade petri dishes (6 cm diameter, Falcon Plastics) were inoculated with $2 \times 10^5$ cells (either H69/P or H69/LX4) in 5 ml of medium on day 0 and drugs added immediately. After 6 days (H69/P) or 7 days (H69/LX4) a count of total phase contrast viable cells per dish was carried out using haemocytometer counting or a total cell count obtained using an electronic cell counter (Coulter Electronics). A comparison between the two counting methods confirmed good agreement.

The times for cell counting were chosen as being near to the end of the exponential growth phase for control cells at which time the original cell number had increased 10-fold. In most experiments, two replicate dishes were used in the control growth and a single dish at each of 6 or 7 different drug doses used to establish the drug response curve. In experiments where duplicate dishes were used for drug treatment groups, close agreement between replicates was seen. Curves were fitted by eye to the drug response data and the values of $ID_{80}$ (the drug dose required to reduce final cell number to 20% of control) read from the curves.

Correspondence: P.R. Twentyman.

Received 16 February 1987; and in revised form, 1 April 1987.

Adriamycin (ADM, Farmitalia) and vincristine (VCR, Eli Lilley) were dissolved in sterile distilled water and added to 5 ml of growth medium in a volume of 50 $\mu$l. Cyclosporins (Cs) A, C, G and H were kindly supplied by Sandoz Ltd (Basle). The structures of these compounds are shown in Figure 1, and their antifugal and immunosuppressive properties summarised in Table I. The cyclosporins were initially dissolved in absolute ethanol. They were then diluted 1:10 in medium and finally added to 5 ml of growth medium in a volume of 50 $\mu$l, thereby producing a final ethanol concentration of 0.1%. This concentration of ethanol does not affect cell growth or drug sensitivity.

Typical response data for H69/P and H69/LX4 cells treated with ADM in the presence or absence of CsA are shown in Figure 2. Data from several experiments where ADM was combined with CsA at a dose of 5 $\mu g\,ml^{-1}$ are shown in Table II and data for VCR combined with CsA in Table III. It can be seen that the addition of CsA at a dose of 5 $\mu g\,ml^{-1}$ led to a small increase in the sensitivity of H69/P to both cytotoxic drugs, whereas a very large increase in the sensitivity of H69/LX4 cells was seen. Sensitisation of H69/LX4 cells to ADM is clearly dependent upon the dose of CsA (Table II) but some sensitisation was still seen at 0.5–1 $\mu g\,ml^{-1}$ CsA.

**Figure 1** Amino acid sequence of cyclosporins. Redrawn from von Wartburg and Traber (1986), $C_9$ is a hitherto unknown amino acid = [2S, 3R, 4R, 6E]-3-hydroxy-4-methyl-2-methyl amino-6-octenoic acid; ABU = L-$\alpha$-amino butyric acid; MEVAL = N-methyl-L-valine; MELEU = N-methyl-L-leucine.

**Table I** Properties of cyclosporins

| *Cyclosporin* | *Antifungal activity* | *Immuno-suppression* |
|---|---|---|
| A | + | +++ |
| C | + | ++ |
| G | + | +++ |
| H | − | − |

From von Wartburg and Traber (1986).

**Figure 2** Number of cells per dish following continuous incubation with various doses of adriamycin. Solid symbols – parent line NCI-H69/P; open symbols – resistant line NCI-H69/LX4. Controls: ●, ○ in presence of cyclosporin A ($1\,\mu g\,ml^{-1}$) △; $2\,\mu g\,ml^{-1}$, ▽; $5\,\mu g\,ml^{-1}$, ■, □.

**Table II** Effect of cyclosporin A upon resistance of NCI-H69 cells to adriamycin

| *Dose of CsA* | *H69/P* | | *H69/LX4* | | |
|---|---|---|---|---|---|
| *($\mu g\,ml^{-1}$)* | *$ID_{80}$($\mu g\,ml^{-1}$)* | *SR* | *$ID_{80}$($\mu g\,ml^{-1}$)* | *SR* | *RF* |
| 0 | 0.0063 | 1.0 | 0.42 | 1.0 | 67 |
| 5 | 0.0045 | 1.4 | 0.022 | 19.1 | 4.9 |
| 0 | 0.0031 | 1.0 | 0.27 | 1.0 | 78 |
| 5 | 0.0023 | 1.3 | <0.011 | >24.5 | <4.8 |
| 0 | 0.0055 | – | 0.80 | 1.0 | 145 |
| 0.5 | – | – | 0.36 | 2.2 | – |
| 1 | – | – | 0.21 | 3.8 | – |
| 2 | – | – | 0.22 | 3.6 | – |
| 5 | – | – | 0.012 | 67 | – |
| 0 | 0.016 | 1.0 | >1.0 | 1.0 | >63 |
| 1 | – | – | 0.82 | >1.2 | – |
| 2 | – | – | 0.19 | >5.2 | – |
| 5 | 0.011 | 1.5 | 0.032 | >31 | 2.9 |

$ID_{80}$ = dose of drug to reduce final cell count to 20% of control.

$$\text{SR (sensitisation ratio)} = \frac{ID_{80} \text{ in absence of CsA}}{ID_{80} \text{ in presence of CsA}}$$

$$\text{RF (resistance factor)} = \frac{ID_{80} \text{ for H69/LX4}}{ID_{80} \text{ for H69/P}}$$

**Table III** Effect of cyclosporin A upon resistance of NCI-H69 cells to vincristine

| *Dose of CsA* | *H69/P* | | *H69/LX4* | | |
|---|---|---|---|---|---|
| *($\mu g\,ml^{-1}$)* | *$ID_{80}$($\mu g\,ml^{-1}$)* | *SR* | *$ID_{80}$($\mu g\,ml^{-1}$)* | *SR* | *RF* |
| 0 | 0.0010 | 1.0 | 2.4 | 1.0 | 1700 |
| 5 | 0.00045 | 2.2 | <0.018 | >133 | <40 |
| 0 | 0.0014 | 1.0 | 1.7 | 1.0 | 1200 |
| 5 | 0.00065 | 2.1 | 0.0080 | 212 | 12.3 |
| 0 | 0.0010 | 1.0 | 0.90 | 1.0 | 900 |
| 1 | 0.00068 | 1.5 | >0.20 | <4.5 | >290 |
| 5 | 0.00048 | 2.1 | 0.0095 | 95 | 19.8 |
| 0 | – | – | 1.6 | 1.0 | – |
| 0.5 | – | – | 1.2 | 1.3 | – |
| 1 | – | – | 0.55 | 2.9 | – |
| 2 | – | – | 0.25 | 6.4 | – |
| 5 | – | – | 0.003 | 470 | |
| 0 | 0.0017 | 1.0 | 2.1 | 1.0 | 1240 |
| 0.5 | – | – | 2.1 | 1.0 | |
| 1 | – | – | 2.0 | 1.1 | |
| 2 | – | – | 1.1 | 2.0 | |
| 5 | 0.00066 | 2.6 | 0.026 | 81 | 39 |

For definitions of $ID_{80}$, SR, RF see **Table II**.

Sensitisation of H69/LX4 to VCR (Table III) was also seen at $1\,\mu g\,ml^{-1}$ of CsA but the effect increases dramatically only between 2 and $5\,\mu g\,ml^{-1}$.

The ability of CsA analogues to overcome ADM resistance is shown in Table IV. Cyclosporin G was at least as active as CsA whilst Cyclosporin C showed less activity at $5\,\mu g\,ml^{-1}$. Cyclosporin H showed relatively little ability to modify ADM resistance even at a dose of $10\,\mu g\,ml^{-1}$.

It should be noted that there is a degree of inter-experiment variability in absolute values of $ID_{80}$. For example, the $ID_{80}$ of ADM alone in line H69/P in the 6 experiments shown in Tables II and IV varies by a factor of 5. We believe that this variability is contributed to by the relative wide spacing of drug doses used (2 fold increments) and probably also the recent culture history of the cells used to set up individual experiments.

These results confirm, in a human small cell lung cancer line, the observations reported by Slater *et al.*, (1986*a*, *b*) that CsA is a highly effective agent in modifying cellular resistance to anthracyclines and VCR. We have also shown that analogues of CsA have a range of abilities to modify resistance. Our studies indicate that there is a clear dose-response relationship between CsA dose and the extent of modification of ADM and VCR resistance. Some effect can

**Table IV** Effect of different cyclosporins upon resistance of NCI/H69 cells to adriamycin

| | *Cyclosporin Dose* | *H69/P* | | *H69/LX4* | | |
|---|---|---|---|---|---|---|
| *Cyclosporin* | *($\mu g\,ml^{-1}$)* | *$ID_{80}$ ($\mu g\,ml^{-1}$)* | *SR* | *$ID_{80}$ ($\mu g\,ml^{-1}$)* | *SR* | *RF* |
| – | 0 | 0.012 | 1.0 | 1.6 | 1.0 | 133 |
| A | 5 | 0.011 | 1.1 | 0.031 | 52 | 2.8 |
| C | 5 | 0.012 | 1.0 | >0.10 | <16 | >8.3 |
| G | 5 | 0.008 | 1.5 | 0.018 | 89 | 2.3 |
| H | 5 | 0.009 | 1.3 | 0.68 | 2.4 | 76 |
| – | 0 | 0.015 | 1.0 | 3.2 | 1.0 | 213 |
| A | 5 | 0.017 | 0.9 | 0.12 | 27 | 7.0 |
| C | 5 | 0.015 | 1.0 | >0.4 | <8 | >27.0 |
| G | 5 | 0.015 | 1.0 | 0.033 | 97 | 2.2 |
| H | 5 | 0.0095 | 1.6 | 2.8 | 1.1 | 294 |
| – | 0 | – | – | 1.4 | 1.0 | – |
| A | 5 | – | – | 0.023 | 61 | – |
| H | 2 | – | – | 0.90 | 1.6 | – |
| H | 5 | – | – | 0.72 | 1.9 | – |
| H | 10 | – | – | 0.54 | 2.6 | – |

For definitions of $ID_{80}$, SR, RF see **Table II**.

be seen at 0.5–1.0 $\mu g\,ml^{-1}$ of CsA, but it requires 5 $\mu g\,ml^{-1}$ to reduce the resistance factor by a factor of 20. Our previous studies using verapamil have shown that a verapamil dose of 6.6 $\mu M$ (3.3 $\mu g\,ml^{-1}$) is required to produce a similar modification of ADM resistance in H69/LX4 (Twentyman *et al.*, 1986*a*). The maximum clinically achievable plasma concentration of verapamil without excessive toxicity is 1–2 $\mu g\,ml^{-1}$ (Rogan *et al.*, 1984), and peak CsA concentrations of 1–2 $\mu g\,ml^{-1}$ are observed following immunosuppressive administration (Kahan *et al.*, 1983). It would therefore appear that CsA is approximately an equal candidate to verapamil for clinical use judged solely on this basis. Examination of the resistance-modifying properties of the 3 CsA analogues indicates a close correlation with their immunosuppressive efficiency. If both of these functions are dependent upon the ability of cyclosporins to inhibit calmodulin acitivity then this is the result that would be expected. Other factors could, however, be involved such as the ability of different cyclosporins to enter the cell. A quite different mechanism of resistance modification has, on the other hand, been proposed by Slater *et al.* (1986*b*) who suggests that CsA may promote cytotoxic drug action at the membrane level by altering the biophysical properties of the plasma membrane. Studies of the effects of cyclosporins upon the cellular pharmacokinetics of ADM and VCR currently in progress in our laboratory should help to elucidate the mechanism of sensitisation.

The adminstration of an immunosuppressive agent to a cancer patient in the hope of overcoming cytotoxic drug resistance is clearly problematic. It is important to determine if analogues of CsA exist which are able to act as resistance modifiers in the absence of immunosuppressive properties. We are currently investigating a range of additional analogues which should help to clarify · the relationship between these properties.

### References

COLOMBANI, P.M., ROBB, A. & HESS, A.D. (1985). Cyclosporin A binding to calmodulin. A possible site of action on T lymphocytes. *Science*, **228**, 337.

KAHAN, B.D., RIED, M. & NEWBURGER, J. (1983). Pharmacokinetics of cyclosporine in human renal transplantation. *Transplant. Proc.*, **15**, 446.

OSIEKA, R., SEEBER, S., PANNEBACKER, R., SOLL, D., GLATTE, P. & SCHMIDT, C.G. (1986). Enhancement of etoposide-induced cytotoxicity by cyclosporin A. *Cancer Chemoth. Pharmacol.*, **18**, 198.

ROGAN, A.M., HAMILTON, T.C., YOUNG, R.C., KLECKER, R.W., JR. & OZOLS, R.F. (1984). Reversal of adriamycin resistance by verapamil in human ovarian cancer. *Science*, **224**, 994.

SLATER, L.M., MURRAY, S.L. & WETZEL, M.W. (1982). Verapamil restoration of daunorubicin responsiveness in daunorubicin-resistant Ehrlich ascites carcinoma. *J. Clin. Invest.*, **70**, 1131.

SLATER, L.M., SWEET, P., STUPECKY, M. & GUPTA, S. (1986*a*). Cyclosporin A reverses vincristine and daunorubicin resistance in acute lymphocyte leukaemia *in vitro*. *J. Clin. Invest.*, **77**, 1405.

SLATER, L.M., SWEET, P., STUPECKY, M., WETZEL, M.W. & GUPTA, S. (1986*b*). Cyclosporin A corrects daunorubicin resistance in Ehrlich ascites carcinoma. *Br. J. Cancer*, **54**, 235.

TSURUO, T., IIDA, H., TSUKAGOSHI, S. & SAKURAI, Y. (1981). Overcoming of vincristine resistance in P388 leukaemia *in vivo* and *in vitro* through enhanced cytotoxicity of vincristine and vinblastine by verapamil. *Cancer Res.*, **41**, 1967.

TSURUO, T., IIDA, H., TSUKAGOSHI, S. & SAKURAI, Y. (1982). Increased accumulation of vincristine and adriamycin in drug-resistant P388 tumour cells following incubation with calcium antagonists and calmodulin inhibitors. *Cancer Res.*, **42**, 4730.

TSURUO, T., IIDA, H., TSUKAGOSHI, S. & SAKURAI, Y. (1983). Potentiation of vincristine and adriamycin effects in human haemopoietic cell lines by calcium antagonists and calmodulin inhibitors. *Cancer Res.*, **43**, 2267.

TWENTYMAN, P.R., FOX, N.E. & BLEEHEN, N.M. (1986a). Drug resistance in human lung cancer cell lines: cross-resistance studies and effects of the calcium transport blocker, verapamil. *Int. J. Radiat. Oncol. Biol Phys.*, **12**, 1355.

TWENTYMAN, P.R., FOX, N.E., WRIGHT, K.A. & BLEEHEN, N.M. (1986b) Derivation and preliminary characterization of adriamycin resistant lines of human lung cancer cells. *Br. J. Cancer*, **53**, 529.

VON WARTBURG, A. & TRABER, R. (1986). Chemistry of the natural cyclosporin metabolites. *Prog. Allergy*, **38**, 28.